# Rexinoids Modulate Effector T Cell Expression of Mucosal Homing Markers CCR9 and α4β7 Integrin and Direct Their Migration *In Vitro*

**DOI:** 10.3389/fimmu.2022.746484

**Published:** 2022-01-27

**Authors:** Kavita R. Manhas, Pamela A. Marshall, Carl E. Wagner, Peter W. Jurutka, Michelle V. Mancenido, Hannah Z. Debray, Joseph N. Blattman

**Affiliations:** ^1^ Biodesign Center for Immunotherapy, Vaccines, and Virotherapy, Arizona State University, Tempe, AZ, United States; ^2^ School of Mathematical and Natural Sciences, Arizona State University West Campus, Glendale, AZ, United States

**Keywords:** migration, mucosal, rexinoids, retinoic acid, retinoid, T-cell, toxicity, vitamin A

## Abstract

Altering T cell trafficking to mucosal regions can enhance immune responses towards pathogenic infections and cancers at these sites, leading to better outcomes. All-*trans*-retinoic acid (ATRA) promotes T cell migration to mucosal surfaces by inducing transcription of the mucosal-homing receptors CCR9 and α4β7 *via* binding to retinoic acid receptors (RARs), which heterodimerize with retinoid X receptors (RXRs) to function. However, the unstable nature and toxicity of ATRA limit its use as a widespread treatment modality for mucosal diseases. Therefore, identifying alternatives that could reduce or eliminate the use of ATRA are needed. Rexinoids are synthetically derived compounds structurally similar to ATRA. Originally named for their ability to bind RXRs, rexinoids can enhance RAR-mediated gene transcription. Furthermore, rexinoids are more stable than ATRA and possess an improved safety profile, making them attractive candidates for use in clinical settings. Here we show that select novel rexinoids act as ATRA mimics, as they cause increased CCR9 and α4β7 expression and enhanced migration to the CCR9 ligand, CCL25 *in vitro*, even in the absence of ATRA. Conversely, other rexinoids act synergistically with ATRA, as culturing cells with suboptimal doses of both compounds resulted in CCR9 expression and migration to CCL25. Overall, our findings show that rexinoids can be used independently or synergistically with ATRA to promote mucosal homing of T cells *in vitro*, and lends support for the prospective clinical use of these compounds in immunotherapeutic approaches for pathogenic infections or cancers at mucosal surfaces.

## Introduction

Mucosal surfaces represent a main entryway for pathogens to anatomic access and are common sites for cancer development. Enhancing immunity at these regions can provide better protection and improve strategies for treating these diseases. Our previous work in mouse models has shown that increasing the migration of vaccinia virus (VACV)-specific memory T cells to mucosal regions during vaccination boosts protection at these sites during VACV challenge (1). Correlative evidence also exists in non-human primate models; in rhesus macaques, the use of an attenuated cytomegalovirus (CMV) vaccine vector for simian immunodeficiency virus (SIV) increases effector T cell numbers at mucosal regions, resulting in vastly improved control and clearance of SIV following viral challenge (2, 3). In humans, clinical evidence further suggests that enhancing immune presence at mucosal sites corresponds positively with protection (4–9). Individuals with vitamin A deficiencies exhibit severely impaired mucosal immunity, resulting in increased susceptibility to infections (10–13). As the heightened immune protection seen is predominantly a result of increased effector T cell presence in the mucosal regions, identifying ways to promote T cell migration to these areas is likely to improve resistance to diseases affecting these areas (1, 14, 15).

Effector T cell trafficking to and entry into mucosal regions is governed by their expression of receptors that instigate mucosal homing, including C-C chemokine receptor type 9 (CCR9) and α_4_β_7_ integrin (α_4_β_7_) (1, 15–17). Upregulation of these mucosal homing receptors during T cell activation is dependent on signaling *via* retinoic acid receptor, a type II nuclear receptor that heterodimerizes with another nuclear receptor, the retinoid X receptor, to mediate transcription (12, 15, 17). Both the RAR and RXR possess α, β, and γ isotypes, with activation of the RARα/RXRα heterodimer implicated in transcription of CCR9 and the α_4_ subunit of α_4_β_7,_ *via* cooperation NFATc2 (12, 15, 16, 18–21). Binding of all-trans retinoic acid (ATRA), a biologically active vitamin A metabolite and the most abundant naturally occurring pan-RAR ligand, to the ligand-binding pocket (LBP) of the RAR triggers activation of the heterodimer, ultimately resulting in RAR-mediated transcription (22–24).

Like the RAR, the RXR also possesses an LBP, and ligand bound to both the RAR and RXR has been shown to enhance transcription of retinoid-dependent genes (18, 23, 25, 26). However, identification of endogenously occurring RXR ligands has remained limited. 9-cis-retinoic acid (9cRA), a naturally occurring stereoisomer of ATRA, has been reported as a high affinity RXR ligand, however its detection *in vivo* remains elusive (27–29). Fatty acids such as docosahexaenoic acid and phytanic acid are also capable of binding the RXR, however endogenously occurring levels are likely too low to activate the receptor under most physiologic scenarios (28, 29). The challenge to conclusively identify naturally occurring RXR ligands has led many groups to utilize synthetic agonists, which have since been coined ‘rexinoids’.

Despite studies showing that ATRA can promote the expression of mucosal T cell homing proteins and subsequent migration to mucosal sites *in vivo*, resulting in better protection against mucosal infection, little is known about the effect of rexinoids on effector T cells. The functional similarity seen between rexinoids and ATRA in experimental models indicates that these synthetic small molecules may be able to exert similar effects as ATRA on effector T cells, by influencing their migration to mucosal-associated regions (30, 31). The ability of rexinoids to bind the RXR suggests that they may improve the impact of endogenous ATRA on T cell mucosal-related function. Additionally, reports that some rexinoids bind to the RAR indicates they may be able to mimic the effect ATRA has on T cell activity (30, 32).

**Figure 1:**
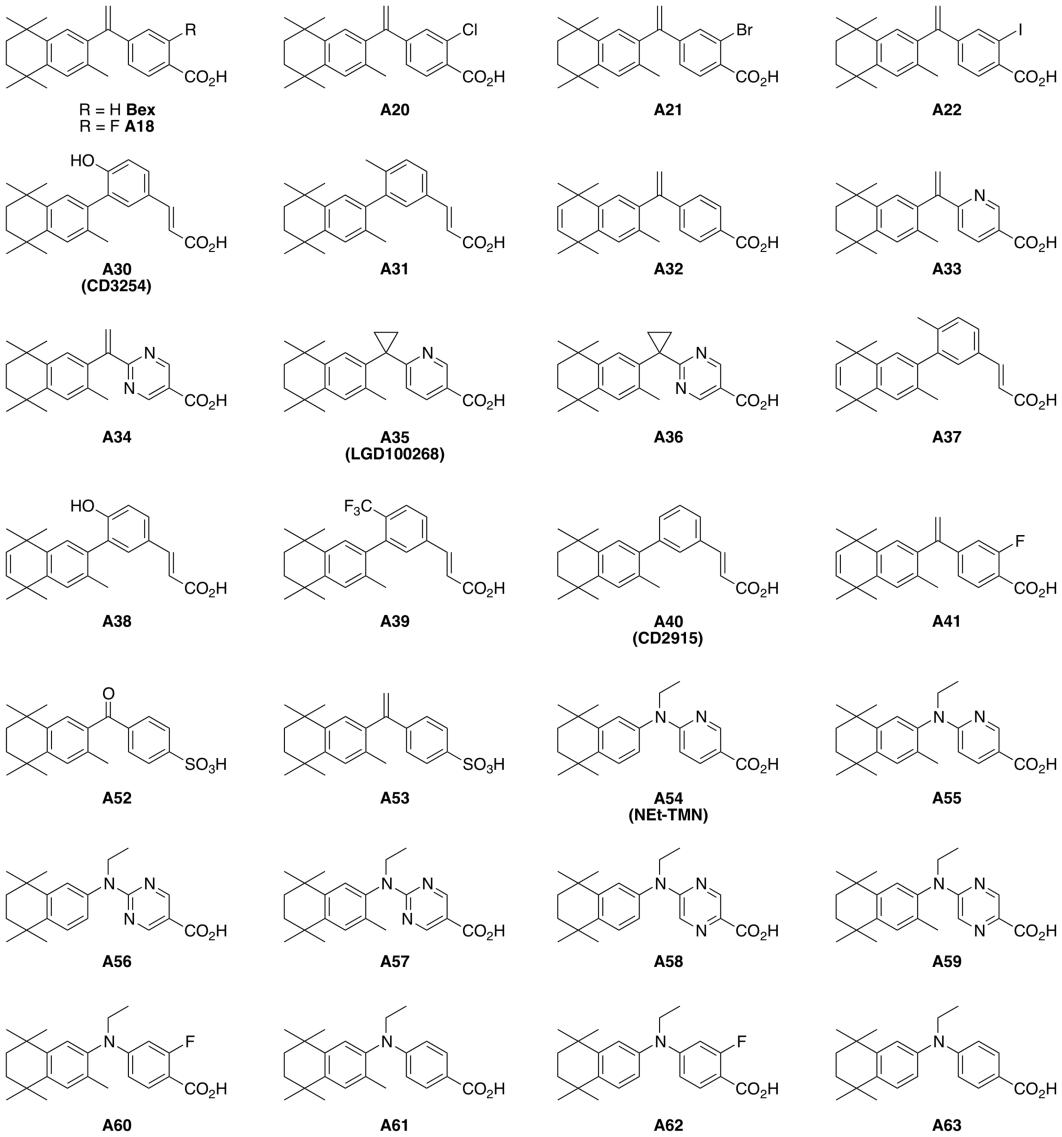
Structures of select rexinoids from the tested panel of rexinoids.

Here we assessed the ability of a panel of rexinoids (**Figure 1**) to induce expression of CCR9 and α_4_β_7_ and to promote T cell migration *in vitro*. These rexinoids include a fluorobexarotene analog, A18, halogenated bexarotene analogs A20-A22, rexinoids A30-A41 which are described in our previous work and references therein, and rexinoids A52-A63 which are again described in our prior work and citations therein (33–36). A subset of rexinoids (A18, A20, and A41) were capable of exerting this effect independently of ATRA, suggesting these compounds can act as ATRA mimics while retaining lower toxicity and enhanced stability. Conversely, other rexinoids (A55, A56, and A57) displayed synergy with suboptimal doses of ATRA to enhance CCR9 expression. Moreover, treatment with ATRA mimics induced T cell migration *in vitro* towards the CCR9 ligand CCL25, while treatment with the ATRA cooperating rexinoids also resulted in improved migration. Furthermore, preliminary *in vivo* data suggest rexinoid treatment is accompanied by reduced toxicity compared to ATRA. Together, these data suggest that rexinoids may have potential to be used as a novel immunotherapeutic treatment modality for mucosal diseases by either replacing ATRA-based strategies or by being used in conjunction with non-toxic ATRA levels to bolster efficacy.

## Materials and Methods

### Rexinoid and ATRA Preparation

A panel of novel rexinoids and bexarotene (BEX) were generously donated by the Wagner, Marshall, and Jurutka labs at 1mM and diluted in 95% ethanol (Koptec) or DMSO (Sigma-Aldrich) to 2x10^5^ nM. Powdered all-trans retinoic acid (Sigma-Aldrich) was dissolved in DMSO and stored in the dark at -20C.

### Lymphocyte Isolation and Culture

Spleens were harvested from B6.Cg-*Tcra^tm1Mom^* Tg(TcrLCMV)327Sdz/TacMmjax (P14), B6.Cg-*Ptprc^a^Pepc^b^* Tg(TcrLCMV)1Aox/PpmJ (SMARTA), or C57BL/6-Tg(TcraTcrb)1100Mjb/J (OT-1) transgenic mice (Jackson Labs) and mechanically dissociated into a single cell suspension using a 70μm nylon mesh strainer (Fisherbrand). All mouse experiments were conducted under institutional care and use committee approval. T cells were stimulated with 1μg/mL of appropriate viral peptide (LCMV gp_33-41_, LCMV gp_61-80_, or OVA_257-264_, respectively) (GenScript; Anaspec). Cells were cultured in a 96-well plate for 8 days using RPMI complete medium (10% FBS, 1% PSG 100X) supplemented with 2.5x10^-5^ μg/μL IL-2 (Gibco) and 100nM of indicated rexinoid treatment or ATRA in a final volume of 200μL. 8 day treatment timeframe was determined using a time course assay (**Supplementary Figure 1**). Fresh culture medium with IL-2 and rexinoid or ATRA was replaced every 48 hours. Vitamin A deficient media was made using charcoal-stripped FBS (ThermoFisher).

### Flow Cytometry

Expression of mucosal homing receptors was determined using flow cytometry. Cells were stained with a 1:100 dilution of the following fluorochrome-conjugated anti-mouse monoclonal antibodies: CCR9 (CW-1.2) and α4β7 (DATK32). P14 cells were further stained with Thy1.1 (HIS51) and CD8α (53-6.7); SMARTA cells were further stained with CD4 (GK1.5) and Vα2 (B20.1); OT-1 cells were further stained with CD8α (53-6.7)and Vα2 (B20.1). All antibodies were purchased from ThermoFisher. Flow cytometry data were collected using a BD LSR Fortessa flow cytometer (BD Biosciences) and analyzed using FlowJo 8.8.7 software. Graphs were created using Prism 8 software (GraphPad). Error bars indicate SD from the mean. Data from SMARTA mice included in Supporting Information.

### *In Vitro* Migration Assay

P14 or SMARTA splenocytes, processed and cultured as described above for 7 days, were plated into the upper chamber of a 96 well HTS Transwell plate insert with 3.0um pore size (Corning) at a concentration of 5x10^5^ cells in 75 μL of chemotaxis buffer (RPMI medium containing 0.1% FBS). Recombinant mouse CCL25/TECK protein (R&D Systems) was reconstituted to 10 μg/mL in 1X PBS (GenClone) containing 0.1% FBS, resuspended in 235 μL chemotaxis buffer at a concentration of 250nM, and plated into the lower chamber. Control wells received no chemokine. Cells were incubated for 6 hours at 37C in 5% CO_2_. Live cells that migrated into the lower chamber were subjected to a 1:2 trypan blue stain and manually quantified using a Neubauer improved C-Chip hemocytometer (INCYTO). Assays using P14 splenocytes were performed in triplicate, while those using SMARTA splenocytes were performed in duplicate. Graphs were created using Prism 8 software. Statistical significance calculated using 2-way ANOVA. Data from SMARTA mice included in Supporting Information.

### *In Vivo* Toxicity

6-12 week old female Balb/cJ mice (Jackson Labs) were inoculated *via* the tail vein with 1x10^6^ K7M2 cells (ATCC; cells not tested for mycoplasma) at day 0, and treated daily for 14 days with either 40mg/kg of vehicle control (n=4), ATRA (n=5), or rexinoid A55 (n=5) delivered *via* intraperitoneal (i.p.) injection, or 100mg/kg vehicle control (n=5), ATRA (n=5), or rexinoid A41 (n=6) delivered *via* oral gavage. Treatment timeframe was determined using previous unpublished data showing lung tumor establishment by Day 14 (data not shown). K7M2 cells were cultured using DMEM complete medium (10% FBS, 1% PSG 100X) under sterile conditions. Vehicle control, ATRA, and rexinoids were dissolved using DMSO and diluted to working concentrations using soybean oil (Sigma-Aldrich). Mouse weights were taken every 24 hours during the course of treatment. For liver toxicity, serum used to measure alanine transaminase (ALT) levels was obtained following cardiac puncture at Day 14, and analyzed using liquid ALT reagent kits (Pointe Scientific). Graphs created using Prism 8 software.

### Statistical Analyses

One-way and two-way Analysis of Variance (ANOVA) were used for data analysis to establish the impacts of rexinoid and/or ATRA on the percentage of CCR9 and α4β7 expression. Follow-up tests for pairwise comparisons among groups were also performed post-ANOVA using Fisher’s Least Significant Difference (LSD) test. All tests were performed at the *α* = 0.05 significance level in JMP Pro 16, a statistical software package.

## Results

### Effector CD8+ T Cells Increase Expression of CCR9 and α_4_β_7_ *In Vitro* Following Rexinoid Treatment

**Figure 2:**
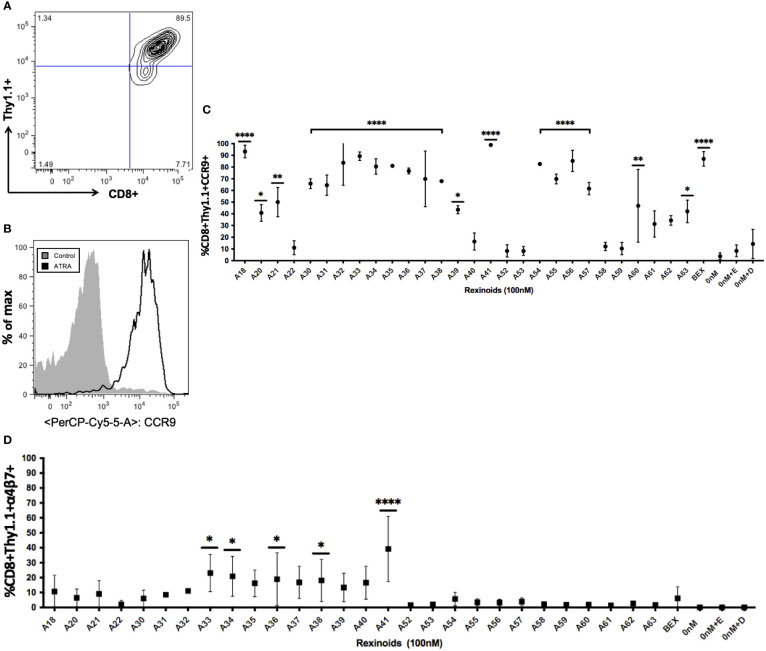
Rexinoid treatment enhances CCR9 and α4β7 expression on effector CD8+ T cells *in vitro*. Splenocytes obtained from a naïve P14 mouse were stimulated with GP_33-41_ peptide and cultured for 8 days with a large panel of novel rexinoids delivered at a 100nM concentration (treatment every 48 hours). Cells were then analyzed for changes in expression of CCR9 and α4β7. Analysis was performed using flow cytometry. **(A)** Antigen-specific effector CD8+ T cells gated using appropriate markers. **(B)** CCR9 expression is upregulated on antigen-specific effector CD8+ T cells following ATRA treatment given over an 8 day time course. **(C)** shows % positive CCR9 expression on antigen-specific effector CD8+ T cells following 8 day rexinoid treatment. Experiment performed in triplicate. **(D)** α4β7 expression on antigen-specific effector CD8+ T cells following 8 day rexinoid treatment. Experiment performed in duplicate. Connecting letters report used to determine statistical significance, with ordered differences report used to compare p-values between groups (* = p < 0.05, ** = p < 0.005, **** = p < 0.0001). All error bars represent SD from the mean.

ATRA is capable of modifying T cell expression of the mucosal homing markers CCR9 and α_4_β_7._ As rexinoids have displayed functional similarity to ATRA in other studies, we sought to determine if our panel of novel rexinoids could also modulate T cell expression of CCR9 and α_4_β_7_. To do this, splenocytes isolated from naïve P14 mice, expressing a transgenic TCR specific for the H-2D^b^ restricted GP_33-41_ peptide of LCMV, were activated *in vitro* and cultured with a panel of 40 rexinoids for 8 days. Many rexinoids administered at 100nM were able to significantly enhance CD8+ T cell expression of CCR9, compared to negative controls (**Figures 2A–C**). Culture with the FDA approved rexinoid bexarotene (BEX) also significantly enhanced CCR9 expression on responding T cells compared to negative controls (**Figure 2C**). Interestingly, rexinoid A41 improved T cell expression of CCR9 better than BEX, identifying a candidate that may possess improved functional efficacy compared to a current existing treatment. Rexinoid treatment also significantly enhanced α_4_β_7_ expression at day 8 of activation compared to negative controls, with A41 again outperforming BEX (**Figure 2D**).

### The Ability of Rexinoids to Enhance CCR9 Expression on Effector T Cells Is Independent of Antigen and MHC Specificity

**Figure 3:**
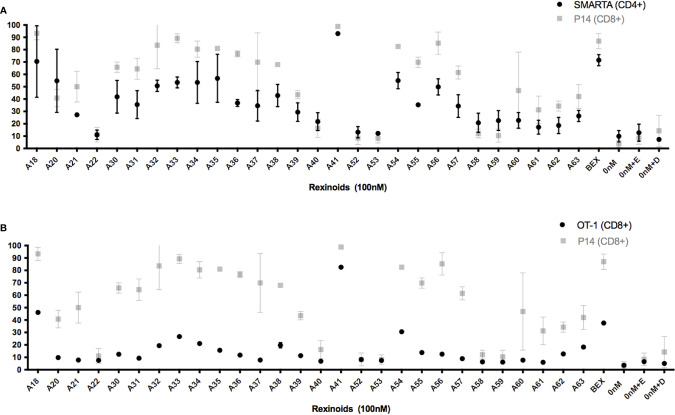
Rexinoid treatment enhances CCR9 expression on effector T cells of different antigen specificity *in vitro*. Splenocytes obtained from naïve SMARTA and OT-1 mice were stimulated with GP_61-80_ peptide and OVA_257-264_, respectively, and cultured for 8 days with the same panel of novel rexinoids delivered at a 100nM concentration (treatment every 48 hours). Cells were then analyzed for changes in expression of CCR9. Analysis was performed using flow cytometry. **(A)** CCR9 expression on antigen-specific effector CD4+ T cells from SMARTA mice following rexinoid treatment (black circles) superimposed onto results from **Figure 2C** (gray squares). SMARTA experiment performed in duplicate. **(B)** CCR9 expression on antigen-specific effector CD8+ T cells from OT-1 mice following rexinoid treatment (black circles) superimposed onto results from **Figure 2C** (gray squares). OT-1 experiment performed in duplicate. All error bars represent SD from the mean.

As rexinoids had a pronounced effect on CCR9 expression, our subsequent experiments focused primarily on the expression of this chemokine receptor as an indicator of mucosal homing protein expression. To determine if the change in T cell expression of CCR9 was antigen or MHC specific, we cultured T cells from either SMARTA and OT-1 mice, TCR transgenic mice expressing TCR specific for different peptide (LCMV GP_61-80_ and OVA_257-264_, respectively) presented in the context of a different MHC (H2-IA^b^ and H-2K^b^, respectively). Rexinoid treatment of T cells from these other TCR transgenic mouse strains also resulted in increased CCR9 expression (**Figures 3A, B**). Moreover, the patterns of increased expression were similar to that obtained for T cells from P14 mice, with no significant differences seen between CD8 and CD4 T cells (p= >0.05). These data suggest that the ability of rexinoids to modulate T cell expression of CCR9 is not limited by antigen specificity or MHC. Moreover, both CD4 and CD8 T cells are able to increase expression of mucosal homing proteins.

### Some Rexinoids Act Independently of ATRA to Enhance T Cell Expression of CCR9

**Figure 4:**
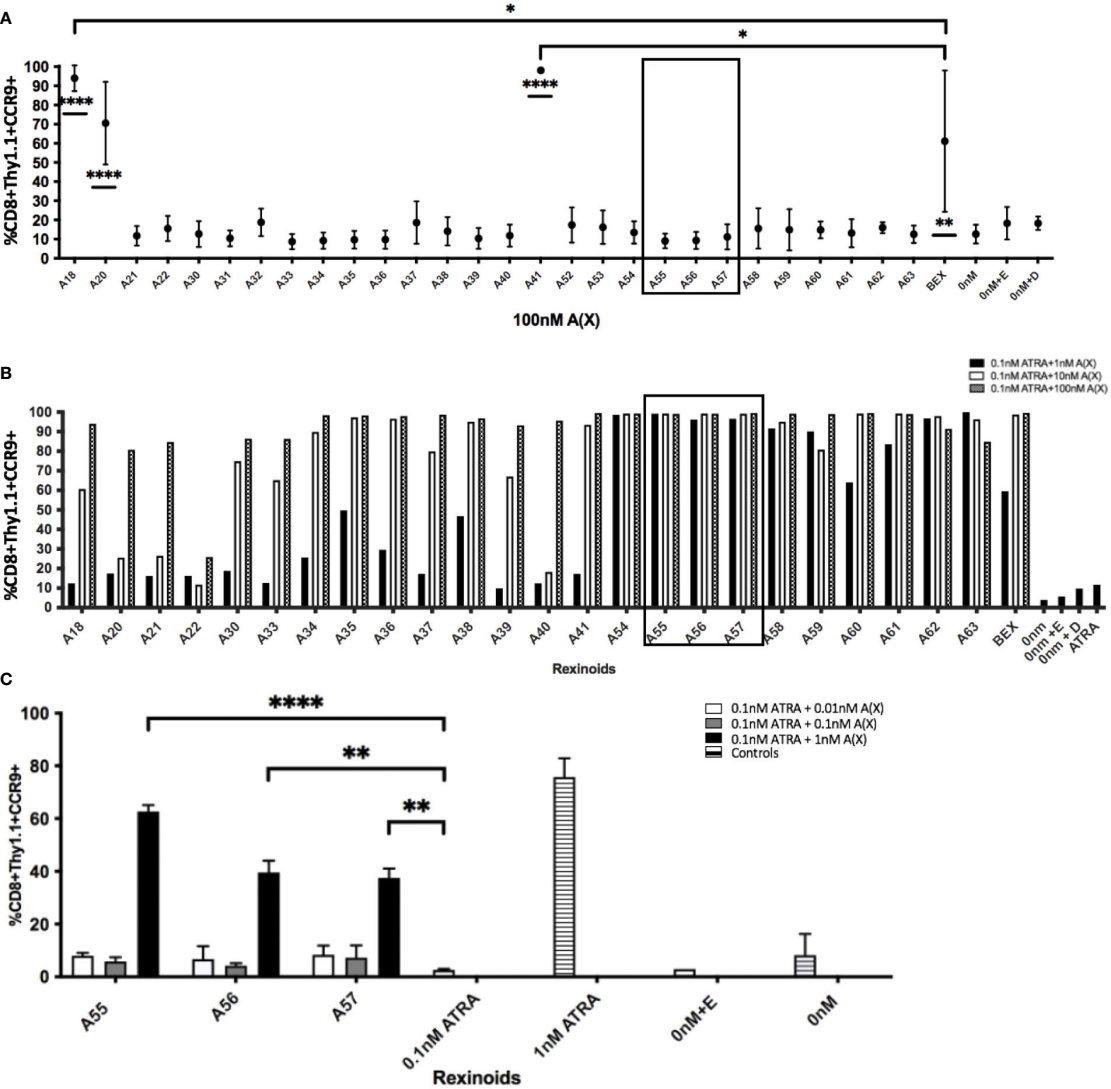
Rexinoids can enhance effector CD8+ T cell expression of CCR9 independently or in combination with ATRA. Splenocytes obtained from P14 mice were stimulated with GP_33-41_ peptide and cultured either with 100nM rexinoids in vitamin A deficient media (top), or in vitamin A deficient media supplemented with suboptimal doses of rexinoids and ATRA. After 8 day culture, effector CD8+ T cells were analyzed for expression of CCR9 using flow cytometry. **(A)** Rexinoids A18, A20, A41 and BEX are able to significantly enhance CCR9 expression independent of ATRA presence, compared to no treatment. Rexinoids A18 and A41 also significantly enhance CCR9 expression, compared to BEX. Experiment performed in triplicate. Connecting letters report used to determine statistical significance, with ordered differences report used to compare p-values between groups (* = p < 0.05, ** = p < 0.005, **** = p < 0.0001). **(B)** Suboptimal doses of several rexinoids cooperate with suboptimal doses of ATRA to enhance CCR9 expression. Boxed region identifies rexinoids that had minimal effect on CCR9 expression when previously delivered at 100nM. (boxed region 3A; graph representative of one experiment). **(C)** Replicate data obtained from culturing cells with suboptimal doses of rexinoid and ATRA. Rexinoids selected were those that showed high cooperativity with ATRA from 3B (boxed region). Suboptimal doses of selected rexinoid combined with suboptimal ATRA significantly improved CCR9 expression, compared to suboptimal ATRA alone (** = p < 0.005, **** = p < 0.0001). Rexinoid dosages lower than 1nM did not result in enhanced CCR9 expression. Experiment performed in triplicate. All error bars represent SD from the mean.

We next sought to determine which rexinoids were capable of altering CCR9 expression independently of ATRA. As charcoal stripping FBS removes lipophilic substances from the serum, including ATRA and other vitamin A derivatives, we supplemented RPMI medium with charcoal stripped FBS in place of standard FBS to create appropriate ATRA deficient culture conditions. P14 T cells were cultured as described above with the indicated rexinoids but without ATRA. The ability of a majority of the rexinoids to alter T cell expression of CCR9 declined to background levels when vitamin A/ATRA was removed from the medium, indicating their dependence on ATRA for increased expression of mucosal homing proteins (**Figure 4A** and **Supplementary Figure 2**). However, some rexinoids (A18, A20, A41) retained their ability to enhance CCR9 expression, despite the lack of vitamin A/ATRA in the culture medium. Overall, these findings demonstrate that select rexinoids can mimic the effects of ATRA in enhancing T cell expression of CCR9, while retaining enhanced safety and stability profiles.

### Some Rexinoids Act Synergistically With ATRA to Enhance T Cell Expression of CCR9

In order to test whether other rexinoids may act synergistically with ATRA, we cultured T cells from P14 mice as described above in charcoal-stripped media that had been supplemented with suboptimal amounts of ATRA (0.1nM) and rexinoids (1nM); neither ATRA nor rexinoids at these concentrations caused expression of CCR9 above background levels (rexinoids only **Figure 4A**, ATRA only **Figure 4B**). T cells cultured with selected rexinoids and ATRA at suboptimal concentrations showed significantly improved expression of CCR9 (boxed region **Figures 4B, C**) compared to treatment with an equivalent dose of ATRA alone (**Figure 4B**) or rexinoid alone (**Figure 4A**). These data suggest that select rexinoids act synergistically with ATRA to promote CCR9 expression. This is supported by previously published *in vitro* data that shows low activation of RAR (**Table 1**). The ability of these rexinoids to cooperate with lower concentrations of ATRA *in vitro* suggests a potential strategy to use these compounds in cooperation with physiological levels of vitamin A levels *in vivo* to promote T cell migration to mucosal regions.

**Table 1:** RXR EC_50_ values in nM and % RAR activation at 100nM selected rexinoids.

Compound	RXR EC_50_ Value (nM) +/- (SD)	RAR % Activation at 100 nM +/- (SD)
A18	43 (5)	25 (6)
A20	90 (14)	13 (2)
A41	71 (10)	48 (10)
A55	13.8 (1.5)	19 (9)
A56	40.9 (0.6)	21 (8)
A57	18.2 (0.4)	16 (6)
BEX	53 (6)	23 (5)

Values obtained from previously published data (32, 34–36). % RAR activation determined from measurements of RAR/RARE reporter activity in transfected cells, rexinoid activity divided by ATRA activity (see ref). Rexinoids included in table were found in this study to either mimic ATRA activity (A18, A20, A41) or cooperate with subtoxic dosages of ATRA (A55, A56, A57) to enhance T cell activity.

### Rexinoid Treatment Improves Chemokine-Mediated Migration of Effector T Cells

**Figure 5:**
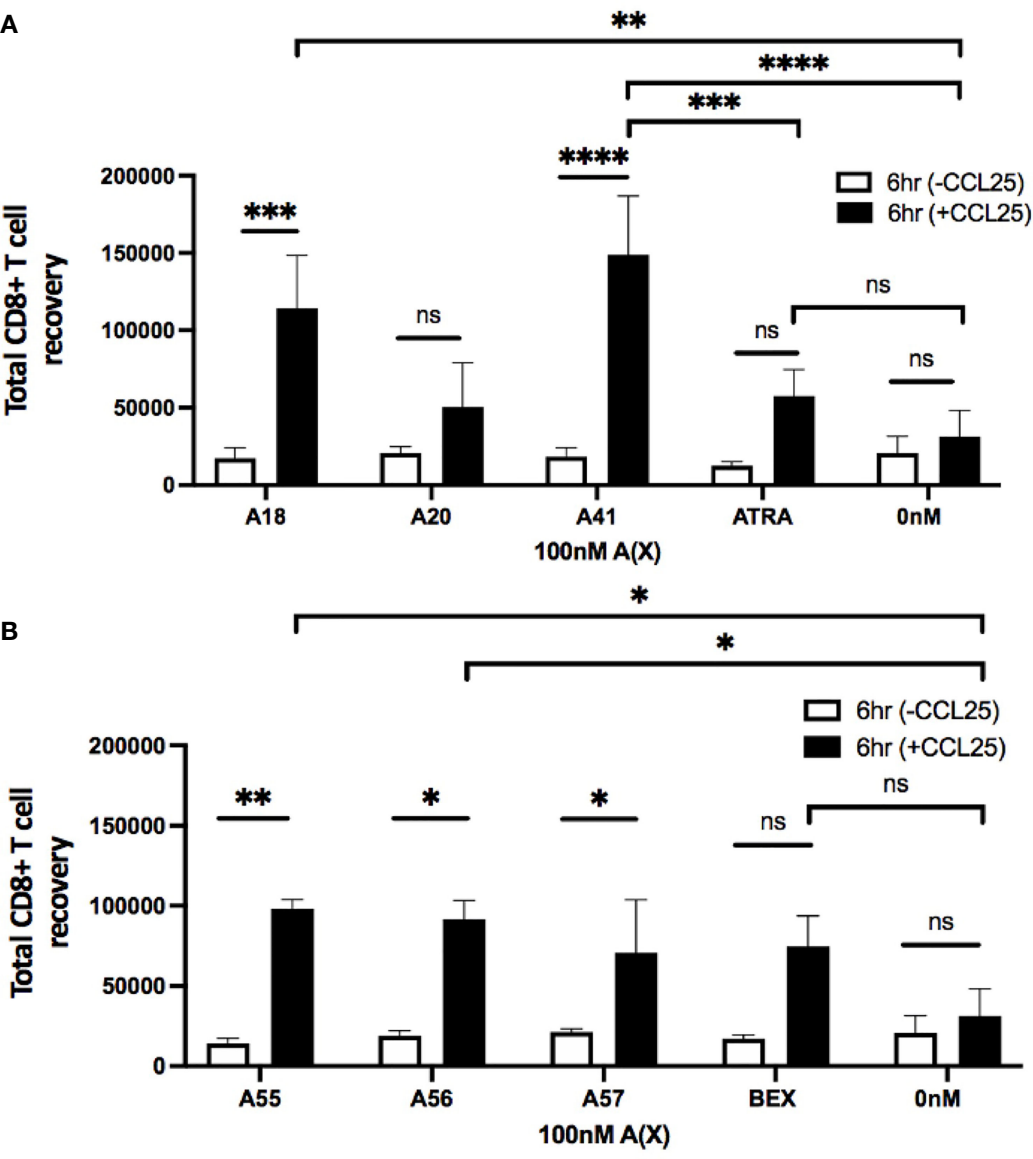
Treatment with ATRA independent and ATRA cooperating rexinoids enhances effector CD8+ T cell migration towards the mucosally expressed chemokine CCL25 *in vitro*. Splenocytes obtained from P14 mice were stimulated with GP_33-41_ peptide and cultured for 7 days with 100nM selected rexinoids or 10nM ATRA. Cells were then subjected to a Boyden chamber assay. 5x10^5^ splenocytes resuspended in chemotaxis buffer were seeded into the top insert of a 96 well HTS Transwell plate and allowed 6 hours to migrate through a membrane (3.0um pore size) towards CCL25 (250nM concentration) plated in the lower chamber. Cells were then isolated from the bottom chamber and manually counted using a hemocytometer. **(A)** Migration following cell culture with ATRA independent rexinoids or ATRA. T cell migration towards CCL25 was significantly improved when cells were cultured with A18 and A41 (adjusted p values = 0.0009 and < 0.0001, respectively). Treatment with A18 or A41 also significantly improved migration towards CCL25 compared to no treatment given (adjusted p values = 0.004 and 0.0001, respectively). Treatment with A41 also significantly improved migration compared to treatment with ATRA (adjusted p value = 0.0008). All ATRA independent rexinoids and ATRA tested in triplicate. **(B)** Migration following cell culture with ATRA cooperating rexinoids or BEX. Migration towards CCL25 was significantly improved when cells were cultured with A55, A56, and A57 (adjusted p values = 0.001, 0.01, and 0.02, respectively). Treatment with A55 or A56 significantly improved migration towards CCL25 compared to no treatment given (adjusted p values = 0.01 and 0.02, respectively). A55, A56, and BEX rexinoids tested in duplicate, A57 rexinoid tested in triplicate. Statistics were calculated using a two-way ANOVA (* = p < 0.05, ** = p < 0.005, *** = p < 0.0005, **** = p < 0.0001). All error bars represent SD from the mean. ns, not significant.

To evaluate the ability of rexinoid-treated T cells to migrate towards chemokine, we performed an *in vitro* transwell migration assay using the CCR9 ligand CCL25. P14 or SMARTA splenocytes were cultured as described above with selected rexinoids that showed the potential to act as an ATRA mimic (A18, A20, A41), or rexinoids that showed synergistic activity with ATRA (A55, A56, A57). Following culture, cells were seeded into the upper well of a transwell plate and incubated to allow for migration through the cell-permeable membrane towards CCL25 in the lower chamber. CD8+ T cells treated with the ATRA mimicking rexinoids A18 and A41 displayed significant migration towards CCL25 (**Figure 5A**). Notably, A41 treatment significantly increased CD8+ T cell migration compared to ATRA treatment (**Figure 5A**). CD8+ T cells treated with the ATRA cooperating rexinoids A55, A56, or A57 also showed significantly better migration towards CCL25 (**Figure 5B**). These results indicate that treatment with ATRA mimicking or ATRA cooperating rexinoids induces effector T cell migration, with some rexinoids outperforming ATRA and BEX. Rexinoid-treated CD4+ T cells displayed increased migration when treated with A41 and A56 (**Supplementary Figure 3**).

### Rexinoid Treatment Displays Lower Toxicity Potential *In Vivo* Compared to ATRA

**Figure 6:**
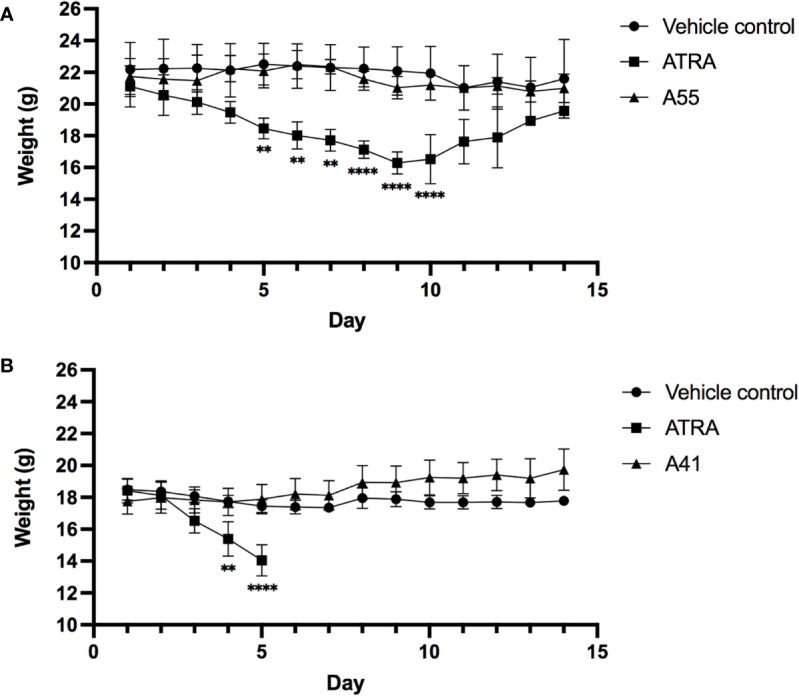
Mice inoculated with K7M2 cells were treated daily with either vehicle control (n=4), rexinoid A55 (n=5), or ATRA (n=4), delivered i.p. at a previously determined concentration of 40mg/kg for 14 days, or with vehicle control (n=5), rexinoid A41 (n=6), or ATRA (n=5) delivered orally at 100mg/kg for 14 days. **(A)** Mice that received A55 treatment i.p. had weight loss similar to negative control mice, while mice that received ATRA treatment displayed significantly larger weight losses during treatment (** = p < 0.005, **** = p < 0.0001). **(B)** Mice that received high dose oral A41 treatment showed slight weight gain, while ATRA-treated mice displayed significant weight loss that necessitated removal from study (** = p < 0.005, **** = p < 0.001). All error bars represent SD from the mean.

To measure the *in vivo* toxicity of rexinoid treatment, we used an established metastatic osteosarcoma (mOS) mouse model for which ATRA has previously been used. Briefly, Balb/cJ mice were inoculated with K7M2 cells *via* tail vein injection prior to daily i.p. treatment with vehicle control or a previously established effective dose of 40mg/kg ATRA or 40mg/kg rexinoid A55. As a measure of toxicity, mouse weights were taken every 24 hours over the course of treatment. Mice that were treated with vehicle control or rexinoid A55 displayed minimal weight changes during the course of treatment, while mice treated with ATRA had significantly higher weight loss (**Figure 6A**), skin erythema, and fur loss (images not shown). *In vivo* toxicity was further examined using a high concentration of treatment delivered orally. Balb/cJ mice were similarly inoculated with K7M2 cells, and treated daily with a predetermined dose of 100mg/kg vehicle control, ATRA, or rexinoid A41, delivered *via* oral gavage. Mice treated with vehicle control or rexinoid A41 displayed minimal weight changes, while ATRA-treated mice displayed significant losses following treatment onset (**Figure 6B**). ATRA-treated mice were removed from study after 5 days treatment, due to rapid physical decompensation. Balb/cJ mice treated with an oral dose of 40mg/kg ATRA also showed greater elevation of the liver enzyme ALT at day 14 compared to mice treated with 40mg/kg vehicle control, A55, or A41 (**Supplementary Figure 4**). Together, these findings suggest that rexinoids are better tolerated and less toxic than ATRA when delivered as a therapeutic modality.

## Discussion

Identifying compounds that can favorably alter T cell migration to mucosal surfaces has the potential to improve immune responses towards diseases at these surfaces. Here we tested a panel of novel rexinoids for their ability to both influence effector T cell expression of mucosal homing markers CCR9 and α4β7 and to affect their migration towards a mucosally expressed chemokine *in vitro*. Our results show that many rexinoids are capable of enhancing CCR9 and α4β7 expression on responding T cells. Several rexinoids induced T cell expression of CCR9 independently, mimicking the naturally occurring biologic ATRA, while others worked synergistically with subtoxic doses of ATRA to enhance expression, indicating a potential to cooperate with vitamin A present *in vivo*. Furthermore, both ATRA mimicking and ATRA cooperating rexinoids were seen to improve T cell migration towards the CCR9 ligand CCL25, with some outperforming bexarotene and ATRA. These findings introduce several rexinoids that can imprint T cells with a mucosal homing phenotype and influence their migration, and may have clinical relevance in treating mucosal diseases.

In addition to CCR9 and α_4_β_7_, the expression of a myriad of other genes have also been shown to be under the control of RAR signaling, including those that inhibit cell cycle progression and promote apoptosis (37–42). These discoveries have led to ATRA being used clinically as an anti-cancer drug; combination treatments that include ATRA have been successful in inducing cancer remission, most notably with acute promyelocytic leukemia (APL), a disease marked by an RARα translocation (43–45). Unfortunately, these favorable results are dampened by adverse side effects attributed to ATRA usage. Various toxicities, including hepatotoxicity due to retinyl ester buildup in hepatic stellate cells (HSCs), and mucocutaneous toxicity, have been reported in cancer patients receiving ATRA treatment (46–49). Here we confirmed that mice treated with ATRA fare poorly, as evidenced by their severe weight loss, physical appearance, and higher ALT levels. An additional complication seen with ATRA use is differentiation syndrome (DS), which can be life-threatening (49–52). Surprisingly, similar adverse health effects have also been reported following the use of synthetic vitamin A derivatives such as isotretinoin and acitretin, which has led us to postulate that the toxicities seen may be due to aberrant activation of the other RAR isoforms (53–55). This is supported by the finding that RARγ deficient mice show resistance to ATRA-mediated toxicity (56). Additionally, activation of all three RAR isoforms have been shown to display teratogenic potential (57). As the ATRA cooperating rexinoids demonstrate high selectivity for the RXR, their use could avoid such toxicity. Furthermore, the widespread use of ATRA is limited due to its instability when exposed to ubiquitous elements such as ultraviolet (UV) light, ambient temperatures, and oxygen (49, 58). The improved stability of rexinoids compared to ATRA is another attractive characteristic; their long shelf life and resistance to fluctuations in temperature, UV light, or oxygen presence makes them more durable treatment options.

In animal models of lung cancer, rexinoid use has been seen to mediate similar antiproliferative and proapoptotic effects on cancer cells as is observed with ATRA (59, 60). Importantly, rexinoid treatment has been shown to be better tolerated than ATRA in both animal and human models. Clinical trial results show that bexarotene, which is currently used as a treatment modality for patients with cutaneous T cell lymphoma (CTCL), can be safely administered at dosages of 300mg/m^2^/day, while side effects are seen with ATRA dosages higher than 45mg/m^2^/day (49, 61–67). However, it is currently unknown for most rexinoids whether they are behaving as ATRA mimics or acting in synergy with ATRA. Here we have not only identified RXR ligands that act similarly to ATRA in altering mucosal homing capabilities, but we have further determined if this effect is dependent on ATRA or not. The rexinoids capable of exerting their effect independently of ATRA have the potential to replace ATRA in therapeutic settings, as they could provide a similar efficacy with a considerably reduced ability to induce toxicity. ATRA cooperating rexinoids also have potential for use in treatment settings; combining these compounds with a much lower dose of ATRA may enhance ATRA mediated effects while minimizing toxicity side effects.

It is well-established that effector T cell infiltration into affected tissues positively correlates with protection from viral infection and tumor regression, therefore identifying methods that can specifically impact their migratory ability may improve immune responses in these microenvironments (1, 14, 68, 69). Our discovery of several rexinoids that favorably modulate T cell mucosal homing abilities *in vitro* indicates that they may be useful as an adjuvant during vaccination towards viruses that infect mucosal surfaces, and in immunotherapies targeting tumors that form at mucosal sites. We have previously shown in mouse models that ATRA has the potential to function as an adjuvant; i.p. injection of ATRA during vaccination increases the number of virus specific T cells to mucosal regions and boosts protection during viral challenge (1). However, this treatment is physically taxing to the mice, resulting in weight loss and inflammation at the injection site. Changing the route of delivery may improve tolerability, however the tradeoff is a reduction in ATRA bioavailability. Our preliminary *in vivo* work has shown that mice are not subject to the same physical discomforts following rexinoid treatment delivered *via* i.p. injection, as observed by their minimal weight loss during treatment (**Figure 6A**). Furthermore, high dose rexinoid delivered orally was well-tolerated, which could compensate for reduced bioavailability when delivered a more preferable route, unlike high dose ATRA (**Figure 6B**). Thus, administering either the ATRA mimicking or ATRA cooperating rexinoids *via* the same route as ATRA may result in similar immune modulating activity, without the associated pathology.

Adoptive cell transfer (ACT) and immune checkpoint blockade (ICB) are immunotherapies currently showing great promise as cancer treatment modalities (70–73). The ability of our rexinoids to modulate T cell migration suggests that their use in tandem with either ACT or ICB therapy may enhance the efficacy of these treatments by directing more effector T cells to tumors at mucosal sites. With ACT, the treatment of *ex vivo* expanded tumor-specific T cells with rexinoids prior to re-infusion can result in more T cells effectively homing to the mucosal tumor, which would result in tumor reduction and possible elimination while avoiding the majority of toxicity issues associated with ATRA use *in vivo*. ICB therapy using a combination of PD-L1 and CTLA-4 blocking antibodies has been shown to reverse tumor-specific effector T cell exhaustion and increase the number of tumor-infiltrating lymphocytes (TILs) present, resulting in improved anti-tumor immune responses (68, 72, 74). Inhibitory interactions between TILs and tumor cells are blocked by anti-PD-L1, while the use of anti-CTLA-4 likely both promotes the activation of new tumor-specific T cells and overcomes regulatory T cell inhibitory pathways. Although promising, this approach currently displays limited efficacy in a subset of patients (69, 75, 76). This may be due to the newly activated T cells ineffectively migrating to the tumor site, resulting in the current TILs becoming overwhelmed, and subsequent re-loss of function. Coupling this ICB approach with our identified rexinoids may ameliorate treatment efficacy towards mucosal cancers by better directing the migration of newly activated tumor-specific T cells to these sites. This would result in larger numbers of functional effector T cells present in the mucosal tumors, resulting in improved cancer control and patient survival.

While this work focuses on immune function resulting from interactions between the RXR and RAR, it should be noted that the RXR is promiscuous. It is an essential partner for a multitude of other receptors, all of which require heterodimeric formation with the RXR to exert their function (26, 77). Rexinoids that did not affect RAR/RXR mediated transcription in terms of CCR9 and α_4_β_7_ expression may play a role in mediating expression of non-immune RAR/RXR dependent genes, or may influence the expression of genes under the control of other RXR heterodimers. The potential of rexinoid treatment to beneficially regulate a variety of biological processes is an exciting and growing research area.

## Data Availability Statement

The authors acknowledge that the data presented in this study must be deposited and made publicly available in an acceptable repository, prior to publication.

## Ethics Statement

All animal studies were reviewed and approved by Arizona State University Institutional Animal Care and Use Committee (IACUC #19-1676R).

## Author Contributions

KM performed experiments, statistical analyses, and wrote the first draft of the manuscript. PM, CW, and PJ contributed to study design and rexinoid synthesis. CW and PJ wrote sections of the manuscript. MM wrote sections of the manuscript and performed statistical analyses. HD performed experiments. JB designed and implemented the study, and wrote sections of the manuscript. All authors read, revised, and approved the manuscript prior to submission.

## Funding

This work was supported in part by funds received from the NIH (NIH SBIR AI089290-01 to JB, NIH R01 AI110720-03 to JB, NIH 1R15CA139364-01A2 to CW, co-Is: PM and PJ, and NIH 1R15CA249617-01 to CW and PJ, co-I: PM) and Arizona State University (ASU GPSA and SOLS RTI funds to KM, Barrett, The Honors College Honors Thesis Reimbursement funds to HD). Patent applications covering the technologies described in this work have been applied for on behalf of the Arizona Board of Regents. This content is solely the responsibility of the authors and does not necessarily represent the official views of the National Institutes of Health.

## Conflict of Interest

The authors declare that the research was conducted in the absence of any commercial or financial relationships that could be construed as a potential conflict of interest.

## Publisher’s Note

All claims expressed in this article are solely those of the authors and do not necessarily represent those of their affiliated organizations, or those of the publisher, the editors and the reviewers. Any product that may be evaluated in this article, or claim that may be made by its manufacturer, is not guaranteed or endorsed by the publisher.

## Glossary

**Table:**

9cRA	9-cis-retinoic acid
α4β7	α4β7 integrin
ACT	Adoptive cell transfer
APL	Acute promyelocytic leukemia
ATRA	All-*trans*-retinoic acid
BEX	Bexarotene
CCL25/TECK	Chemokine ligand 25/Thymus-expressed chemokine
CCR9	C-C chemokine receptor 9
CD4	Cluster of differentiation 4
CD8	Cluster of differentiation 8
CMV	Cytomegalovirus
CTCL	Cutaneous T-cell lymphoma
CTLA-4	Cytotoxic T lymphocyte antigen 4
DMEM	Dulbecco’s Modified Eagle Medium
DMSO	Dimethylsulfoxide
DS	Differentiation syndrome
FBS	Fetal bovine serum
HSC	Hepatic stellate cell
ICB	Immune checkpoint blockade
i.p.	intraperitoneal
LBP	Ligand binding pocket
LCMV	Lymphocytic choriomeningitis virus
MHC	Major histocompatibility complex
mOS	Metastatic osteosarcoma
PD-L1	Programmed death-ligand 1
RAR	Retinoic acid receptor
RPMI	Roswell Park Memorial Institute Medium
RXR	Retinoid X receptor
SIV	Simian immunodeficiency virus
TCR	T-cell receptor
TIL	Tumor infiltrating lymphocyte
UV	Ultraviolet
VACV	Vaccinia virus
