# Supplementary material for: Rexinoids Modulate Effector T Cell Expression of Mucosal Homing Markers CCR9 and α4β7 Integrin and Direct Their Migration In Vitro
Source: Front Immunol. 2022 Jan 27;13:746484. doi: 10.3389/fimmu.2022.746484 (PMC8829570; doi:10.3389/fimmu.2022.746484)
Supplement: Supplementary file 1 [file DataSheet_1.docx]

**Supplementary Figure 1**.**
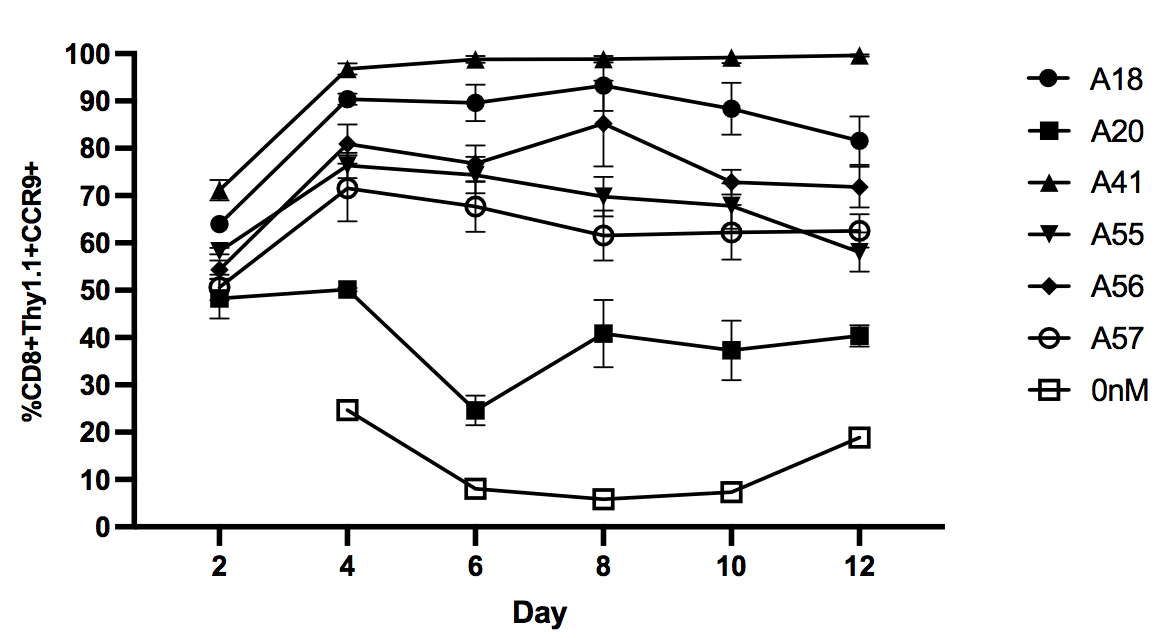
** **Time course assay shows optimal CCR9 expression at Day 8.** Splenocytes obtained from naïve P14 mice were stimulated with GP_33-41_ peptide and cultured with 100nM select rexinoids (key to right). Flow cytometric analysis of CCR9 expression was performed every 2 days. A majority of rexinoids stimulated cells to optimally express CCR9 by day 8, which corresponds with the peak of primary murine immune responses.

**(A)**

**
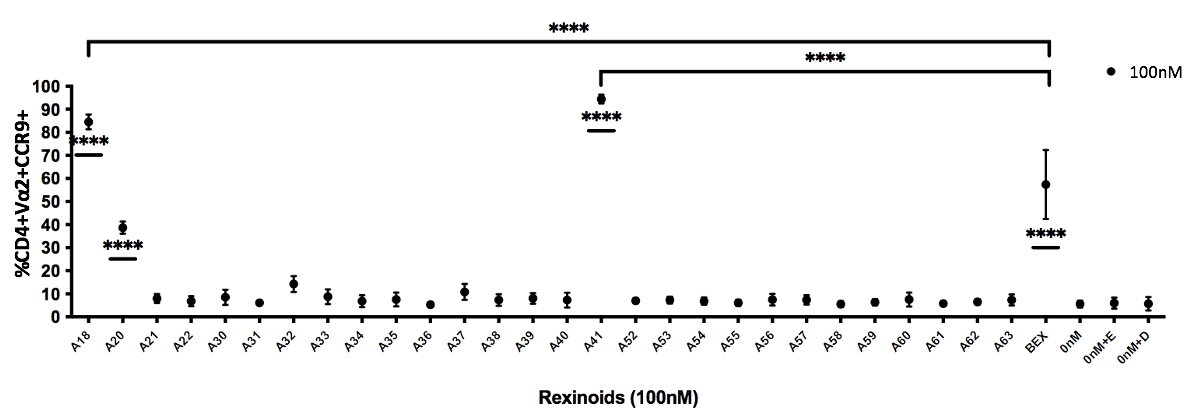
Supplementary Figure 2**. **Rexinoids can enhance effector CD4+ T cell expression of CCR9 independently of ATRA.** Splenocytes obtained from naïve SMARTA mice were stimulated with GP_61-80_ peptide and cultured with 100nM rexinoids in vitamin A deficient media. After 8 day culture, effector CD4+ T cells were analyzed for expression of CCR9 using flow cytometry. **(A)** Rexinoids A18, A20, A41 and BEX are able to significantly enhance CCR9 expression independent of ATRA presence compared to no treatment, similar to their activity on effector CD8+ T cells. Rexinoids A18 and A41 also significantly enhance CCR9 expression compared to BEX. Experiment performed in triplicate. Connecting letters report used to determine statistical significance, with ordered differences report used to compare p-values between groups (**** = p<0.0001).Error bars represent SD from the mean.

**(A)**

**
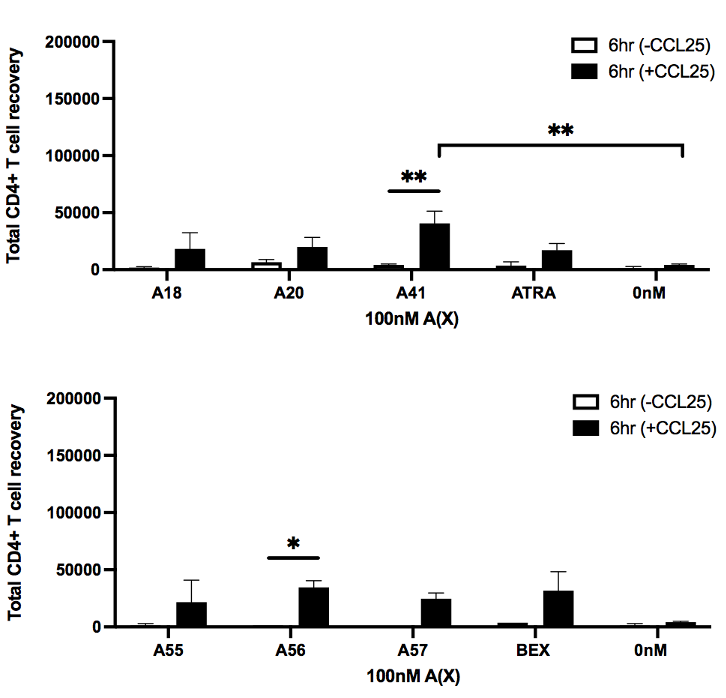
**

**(A)**

**(B)**

**Supplementary Figure 3. Treatment with ATRA independent and ATRA cooperating rexinoids can enhance effector CD4+ T cell migration towards the mucosally expressed chemokine CCL25 *in vitro*.** Splenocytes obtained from SMARTA mice were stimulated with GP_61-80_ peptide and cultured for 7 days with 100nM selected rexinoids or 10nM ATRA. Cells were then subjected to a Boyden chamber assay, as described in Figure 4 legend. **(A)** Migration following cell culture with ATRA independent rexinoids or ATRA. T cell migration towards CCL25 was improved when cells were cultured with rexinoids A18, A20, A41, and BEX. Migration was significantly improved when cells were cultured with A41 in the presence of CCL25 (adjusted p value = 0.006). Rexinoid treatment also improved migration towards CCL25 compared to no treatment given, with significantly improved migration seen following A41 treatment (adjusted p value = 0.006). Treatments tested in duplicate. **(B)** Migration following cell culture with ATRA cooperating rexinoids or BEX. Migration towards CCL25 was improved when cells were cultured with A55, A56, A57, and BEX, with significantly improved migration seen following A56 treatment (adjusted p value = 0.04). Treatments tested in duplicate. Statistics were calculated using a two-way ANOVA (* = p <0.05, ** = p<0.005). Error bars represent SD from the mean.


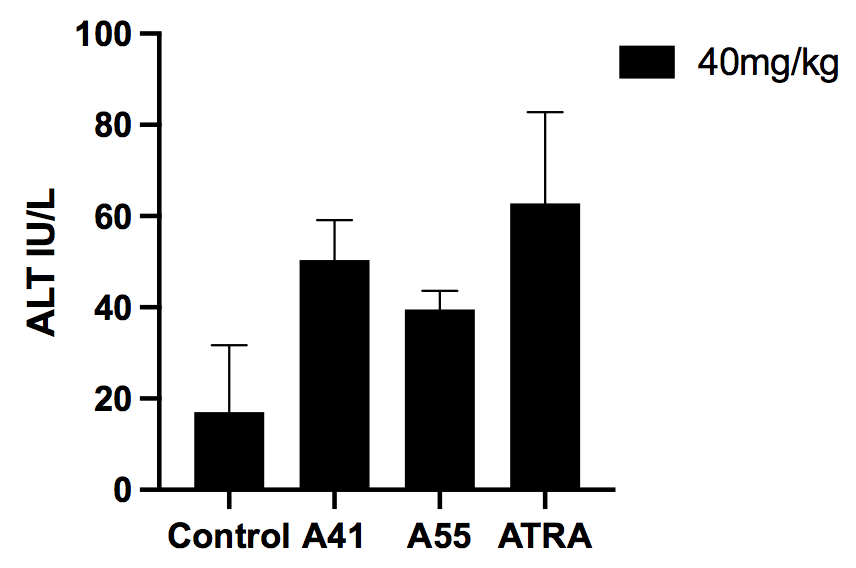


**Supplementary Figure 4. ATRA-treated mice show higher levels of ALT.** Balb/cJ mice were treated orally with 40mg/kg vehicle control (n=2), A41 (n=2), A55 (n=2), or ATRA (n=2) for two weeks, with serum obtained at day 14 to measure for levels of the liver enzyme ALT. Mice treated with ATRA showed higher levels of ALT compared to other groups, however these differences were not seen to be significant.
